# Oral intake of rice overexpressing ubiquitin ligase inhibitory pentapeptide prevents atrophy in denervated skeletal muscle

**DOI:** 10.1038/s41538-021-00108-0

**Published:** 2021-09-09

**Authors:** Reiko Nakao, Weilin Shen, Yasuka Shimajiri, Kumiko Kainou, Yuki Sato, Anayt Ulla, Kohta Ohnishi, Miyuki Ninomiya, Ayako Ohno, Takayuki Uchida, Mitsuru Tanaka, Kazuhito Akama, Toshiro Matsui, Takeshi Nikawa

**Affiliations:** 1 Department of Nutritional Physiology, Institute of Biomedical Sciences, Tokushima University Graduate School, Tokushima, Japan; 2 Department of Bioscience and Biotechnology, Faculty of Agriculture, Graduate School, Kyushu University, Fukuoka, Japan; 3 Faculty of Life and Environmental Science, Shimane University, Matsue, Shimane Japan; 4 EditForce, Fukuoka, Japan; 5 Department of Clinical Nutrition and Food Management, Institute of Biomedical Sciences, Tokushima University Graduate School, Tokushima, Japan

**Keywords:** Mass spectrometry, Physiology

## Abstract

We previously reported that intramuscular injections of ubiquitin ligase CBLB inhibitory pentapeptide (Cblin; Asp-Gly-pTyr-Met-Pro) restored lost muscle mass caused by sciatic denervation. Here, we detected Cblin on the basolateral side of Caco-2 cells after being placed on the apical side, and found that cytochalasin D, a tight junction opener, enhanced Cblin transport. Orally administered Cblin was found in rat plasma, indicating that intact Cblin was absorbed in vitro and in vivo. Furthermore, transgenic Cblin peptide-enriched rice (CbR) prevented the denervation-induced loss of muscle mass and the upregulation of muscle atrophy-related ubiquitin ligases in mice. These findings indicated that CbR could serve as an alternative treatment for muscle atrophy.

## Introduction

Insulin-like growth factor 1 (IGF-1) signaling is a major pathway of skeletal muscle growth and hypertrophy^1^. We previously reported that skeletal muscle disuse such as that associated with space flight, tail suspension, and sciatic denervation upregulates expression of the *Cblb* gene encoding the ubiquitin ligase, Casitas B-lineage lymphoma b (CBLB)^2^. Overexpressed CBLB protein induces the ubiquitination and degradation of insulin receptor substrate 1 (IRS1), the key adapter protein of IGF-1 signaling, in vitro and in vivo^3,4^, then induces the expression of genes encoding muscle atrophy-related ubiquitin ligases, such as muscle atrophy F-box protein (MAFbx)^5,6^ and muscle RING finger 1 (MuRF1)^5^ by activating forkhead transcription factor 3 (FOXO3)^7,8^. We also showed that *Cblb*-deficient mice are resistant to muscle atrophy induced by tail suspension^4^. These results indicated that CBLB-dependent IRS1 degradation is a critical mediator of muscle atrophy under unloading conditions.

We developed a CBLB inhibitory peptide (Cblin peptide) with the amino acid sequence Asp-Gly-phosphorylated (p) Tyr-Met-Pro to prevent muscle atrophy. This peptide mimics the sequence of tyrosine^608^-phosphorylated IRS1, interacts with the tyrosine kinase binding (TKB) domain of CBLB, and subsequently inhibits the CBLB-mediated ubiquitination of IRS1^9^. Injecting Cblin peptide into the skeletal muscle of mice with sciatic denervation prevented the loss of muscle mass^4^. However, repetitive intramuscular injections of the peptide are invasive and stressed the mice.

Many di- and tripeptides derived from food sources exert bioactivities such as antihypertensive effects in vivo^10–12^. Bioactive di- and tripeptides are absorbed into the bloodstream via the intestinal proton-coupled peptide transporter-1, PEPT1, encoded by the *Slc15a1* gene^13,14^. A peptide-binding cavity in PEPT1 allows it to attach to di- and tripeptides, but not to peptides with ≥4 amino acid residues. The orally administered pentapeptide, Arg-Val-Pro-Ser-Leu, which has angiotensin-converting enzyme inhibitory activity in vitro lowers blood pressure in spontaneously hypertensive rats^15^. Oligopeptides such as Arg-Val-Pro-Ser-Leu, Val-Leu-Pro-Val-Pro^16^, and Thr-Asn-Gly-Ile-Ile-Arg^17^ can penetrate Caco-2 cell monolayers, presumably via paracellular tight junctions. We previously discovered that the respective tetra- and pentapeptides Gly-Sar-Sar-Sar and Gly-Sar-Sar-Sar-Sar that have methylated peptide bonds can be absorbed intact in rats, although to a lesser extent than di- and tripeptides^18^. However, whether Cblin peptide is similarly absorbed requires further investigation.

We previously showed that feeding mice with soy glycinin inhibited muscle atrophy induced by sciatic denervation^19^. We speculated that this effect might have resulted from proteases in the gastrointestinal tract releasing Cblin-like peptides such as Asp-Ile-Tyr-Asn-Pro from soy glycinin. We considered that consuming foods containing Cblin-like peptides might prevent muscle atrophy. Akama et al. developed a transgenic rice strain that is rich in gamma-aminobutyric acid^20^, and novel plant systems that stably accumulate Asp-Gly-Tyr-Met-Pro in rice seeds^21^. The present study aimed to determine whether Asp-Gly-Tyr-Met-Pro is absorbed by the intestine and whether Cblin-rich rice (CbR) can prevent muscle atrophy in rodents.

## Results and discussion

### Transport of Cblin peptides across Caco-2 cell monolayers

**Fig. 1:**
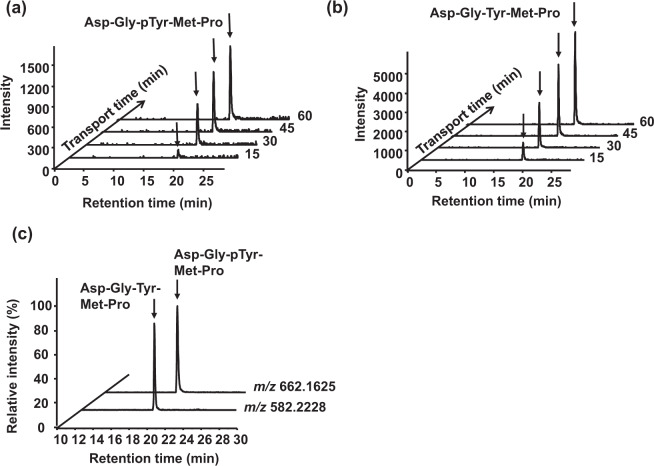
Transport of Cblin pentapeptides in Caco-2 cells. Chromatograms show apical to basolateral transepithelial transport of 1 mM Asp-Gly-pTyr-Met-Pro (**a**) and Asp-Gly-Tyr-Met-Pro (**b**) in Caco-2 cell monolayers over 60 min. Samples from the basolateral side at 15, 30, 45, and 60 min were analyzed using LC–TOF/MS with [M + H]^+^ at *m*/*z* 662.1625 for Asp-Gly-pTyr-Met-Pro and 582.2228 for Asp-Gly-Tyr-Met-Pro. (**c)** Dephosphorylation of Asp-Gly-pTyr-Met-Pro monitored in basolateral solution after 60 min of transport by extracting ions with *m*/*z* 582.2228 corresponding to Asp-Gly-Tyr-Met-Pro ions.

The intestinal transport of Cblin peptides Asp-Gly-pTyr-Met-Pro and Asp-Gly-Tyr-Met-Pro (initial apical concentration: 1 mM) was investigated in Caco-2 cell monolayers with transepithelial electrical resistance (TEER) > 300 Ω · cm^2^ over a period of 60 min. Basolateral solutions were sampled every 15 min for 60 min and analyzed by LC–TOF/MS. The mass spectrometry (MS) signals ([M + H]^+^) corresponding to 662.1625 *m*/*z* for Asp-Gly-pTyr-Met-Pro and 582.2228 *m*/*z* for Asp-Gly-Tyr-Met-Pro were time dependently increased at retention times (RTs) of 19.8 and 19.4 min, respectively (Fig. 1a, b). Therefore, both peptides penetrated Caco-2 cell monolayers and remained intact. Peptide fragments were screened based on their specific *m*/*z* values. After 60 min, Asp-Gly-pTyr-Met-Pro was dephosphorylated and Asp-Gly-Tyr-Met-Pro was transported to the basolateral solutions at an estimated concentration that was similar to that of intact Asp-Gly-pTyr-Met-Pro (Fig. 1c). Density-enhanced protein phosphatase-1 or intestinal alkaline phosphatase expressed on the surface of epithelial cells or released into media might be involved in the dephosphorylation process^22,23^. No other peptide fragments were found, indicating that both peptides are resistant to peptidase degradation in Caco-2 cells (data not shown). The calculated apparent permeability coefficients (*P*_app_) were 3.5 ± 1.2 × 10^−7^ and 7.0 ± 0.8 × 10^−7^ cm/s for Asp-Gly-pTyr-Met-Pro and Asp-Gly-Tyr-Met-Pro, respectively. These values were in the same order as the pentapeptide transport model Gly-Sar-Sar-Sar-Sar (*P*_app_: 8.6 ± 0.6 × 10^−7^ cm/s^24^; Table 1), indicating that the transport velocity of Cblin peptides across Caco-2 cell monolayers was appropriate.

**Table 1:** Apparent permeability coefficient (*P*_app_) of Asp-Gly-pTyr-Met-Pro and Asp-Gly-Tyr-Met-Pro in Caco-2 cell monolayers.

	*P*_app_ (×10^−7^ cm/s)
Asp-Gly-pTyr-Met-Pro	3.5 ± 1.2^b^
Asp-Gly-Tyr-Met-Pro	7.0 ± 0.8^ab^
Gly-Sar-Sar-Sar-Sar	8.6 ± 0.6^a,^*

Data are expressed as means ± SEM (*n* = 4–6). Different superscript letters indicate statistical differences (*P* < 0.05).

^*^P_app_ is described in ref. ^24^.

**Fig. 2:**
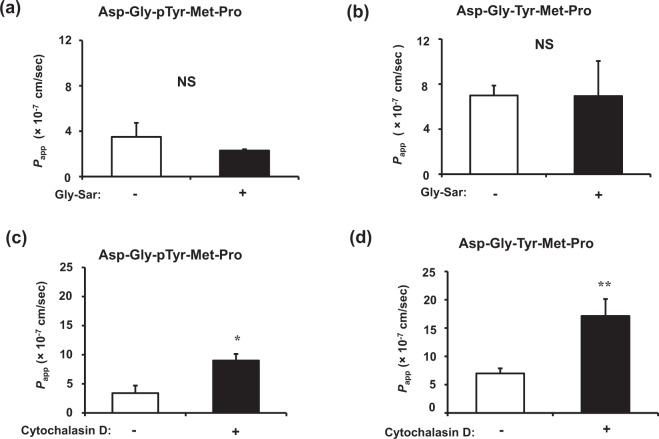
Effects of Gly-Sar and cytochalasin D on transport of Cblin pentapeptides in Caco-2 cells. Effects of 10 mM Gly-Sar on transport of 1 mM Asp-Gly-pTyr-Met-Pro (**a**) and Asp-Gly-Tyr-Met-Pro (**b**) across Caco-2 cell monolayers for 60 min. Basolateral peptide concentrations were determined after 60 min by LC–TOF/MS. Effects of cytochalasin D on transport of 1 mM Asp-Gly-pTyr-Met-Pro (**c**) and Asp-Gly-Tyr-Met-Pro (**d**) across Caco-2 cell monolayers for 60 min. Caco-2 cell monolayers were incubated with or without cytochalasin D (0.5 μg/mL) for 30 min. Basolateral peptide concentrations were determined by LC–TOF/MS. Values are expressed as means ± SEM, *n* = 4–6. ^*^P < 0.05, ^**^P < 0.01. NS, not significant.

Transport pathway studies showed that the *P*_app_ values of Asp-Gly-pTyr-Met-Pro and Asp-Gly-Tyr-Met-Pro (Fig. 2a, b) were not affected by 10 mM Gly-Sar, a PEPT1 substrate, suggesting that PEPT1 is not involved in transporting the two pentapeptides. These results concurred with those of a study showing that PEPT1 only recognizes and transports peptides with ≤3 amino acid residues^25^. Meanwhile, the *P*_app_ values of both peptides were significantly higher in monolayers incubated with cytochalasin D, a paracellular tight junction opener, than in untreated membranes (Fig. 2c, d). These results indicated that Cblin passively diffuses across paracellular tight junctions like several other oligopeptides^26^. We could not rule out the involvement of other pathways in the intestinal absorption of Cblin pentapeptides. The transcytosis mechanism is involved in transporting the decapeptide Tyr-Trp-Asp-His-Asn-Asn-Pro-Gln-Ile-Arg that is derived from rapeseed protein^27^ and β-casein, a 17-residue peptide (193–209)^28^. Chothe et al. reported that some transport systems in intestinal epithelial cell lines might be specific to oligopeptides with >3 residues^29^.

### Absorption of Asp-Gly-Tyr-Met-Pro into rat circulation

**Fig. 3:**
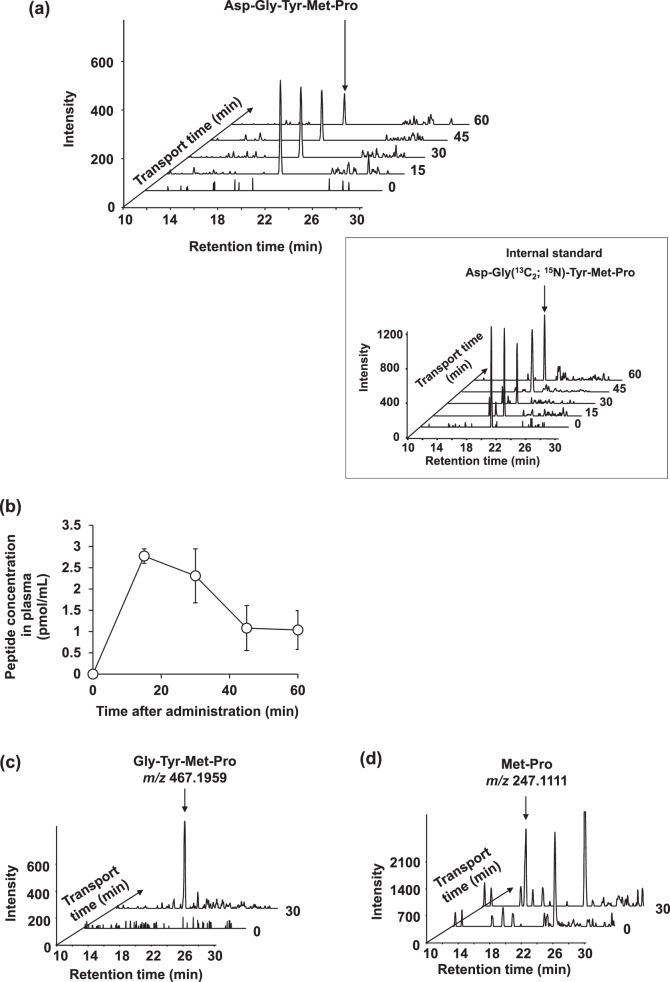
Absorption of Asp-Gly-Tyr-Met-Pro in rat plasma. **a** Absorption of Asp-Gly-Tyr-Met-Pro in 8-week-old Sprague-Dawley rats. Stacked intensity-time chromatograms of Asp-Gly-Tyr-Met-Pro with [M + H]^+^ of *m*/*z* 582.2228 and 50 pmol/mL of labeled internal standard (IS) Asp-Gly(^13^C_2_; ^15^N)-Tyr-Met-Pro ([M + H]^+^: *m*/*z* 585.2228) (insert) created by sampling plasma at 0, 15, 30, 45, and 60 min from tail veins of rats dosed with 100 mg/kg BW Asp-Gly-Tyr-Met-Pro. **b** Time course of appearance of Asp-Gly-Tyr-Met-Pro in plasma of rats orally administered with 100 mg/kg BW determined by LC–TOF/MS. Data are expressed as means ± SEM (*n* = 5). **c**, **d** Extracted ions corresponding to those of possible metabolites, Gly-Tyr-Met-Pro ([M + H]^+^: *m*/*z* 467.1959) and Met-Pro ([M + H]^+^: *m*/*z* 247.1111) were detected in rat plasma at 30 min after administration.

Sprague-Dawley (SD) rats were fasted for 16 h, then administered p.o. with 100 mg/kg of body weight (BW) of Asp-Gly-Tyr-Met-Pro, and blood was sampled at 0, 15, 30, 45, and 60 min thereafter. Absolute plasma Asp-Gly-Tyr-Met-Pro concentrations were determined using an internal standard comprising plasma spiked with a final concentration of 50 pmol/mL Asp-Gly-Tyr-Met-Pro labeled with stable isotopes (Asp-Gly[^13^C_2_; ^15^N]-Tyr-Met-Pro) (Fig. 3a, box). A peak corresponding to *m*/*z* of the Asp-Gly-Tyr-Met-Pro peak on MS was undetectable in plasma at 0 min (Fig. 3a). Absorbed Asp-Gly-Tyr-Met-Pro peaked at 2.78 ± 0.17 pmol/mL in plasma samples at 15 min (Fig. 3b and Table 2), then decreased over the next 45 min (Fig. 3a, b). The pentapeptide at 50 and 200 mg/kg BW was also absorbed (Supplementary Table 1). We previously showed that the orally administered pentapeptide, Gly-Sar-Sar-Sar-Sar, is absorbed into rat circulation^18^. Although less Asp-Gly-Tyr-Met-Pro than Gly-Sar-Sar-Sar-Sar was absorbed, the model pentapeptide was more resistant to peptidase degradation due to having methylated peptide bonds^18^. Therefore, we screened plasma samples for peptide fragments generated by enzymatic degradation based on *m*/*z* values and found tetra- and dipeptide metabolites corresponding to the *m*/*z* values of Gly-Tyr-Met-Pro and Met-Pro, respectively (Fig. 3c, d). Exopeptidases such as aminopeptidase and peptidyl dipeptidase expressed on the brush border membrane might have been responsible for generating these peptide fragments^30^. Other metabolites such as amino acids and hydrophilic peptides might not have been retained on reversed phase columns under our experimental conditions. The bioavailability of Asp-Gly-Tyr-Met-Pro and its metabolism require further clarification.

**Table 2:** Pharmacokinetics after oral administration of Asp-Gly-Tyr-Met-Pro to 8-week-old Sprague-Dawley rats.

Measured parameters	Oral dose (100 mg/kg BW)
*C*_max_ (pmol/mL plasma)	2.78 ± 0.17
*t*_max_ (min)	15
AUC_0–60 min_ (pmol/min/mL plasma)	100.35 ± 23.40
*t*_1/2_ (min)	28

Data are expressed as means ± SEM (*n* = 5).

AUC_0–60 min_, area under plasma concentration vs. time curve; BW, body weight; *C*_max_, maximum plasma concentration; *t*_1/2_, elimination half-life; *t*_max_, time to reach peak plasma concentration.

### Characterization of Cblin peptide-enriched rice (CbR)

**Fig. 4:**
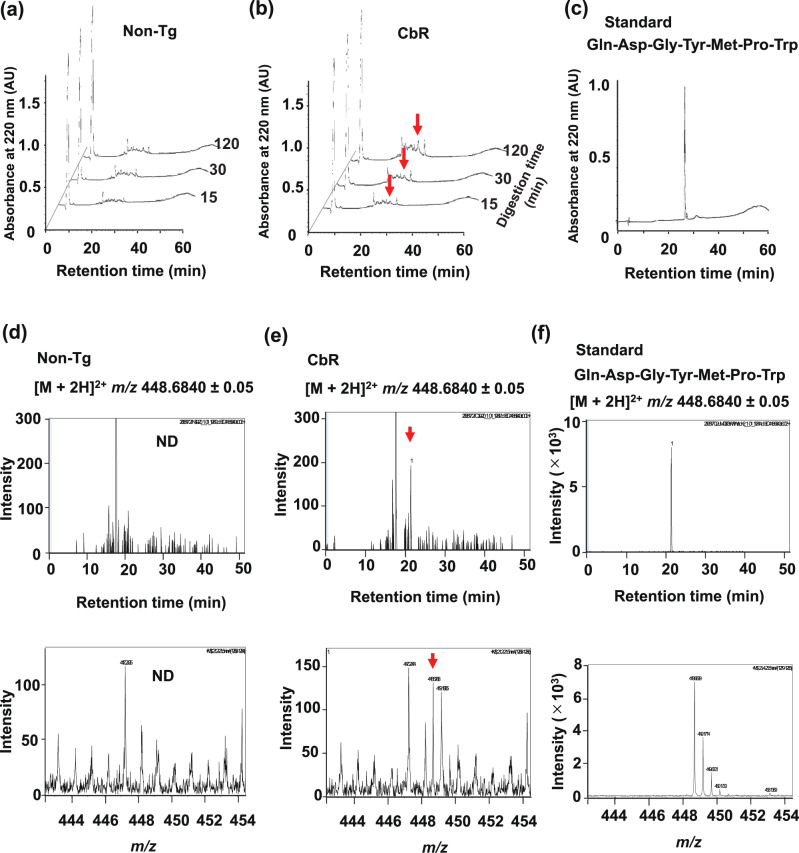
Chymotrypsin hydrolysates of non-Tg and CbR determined by HPLC. Chromatograms of chymotrypsin-digested glutelin-derived peptides from nontransgenic (non-Tg; **a**) or Cblin-enriched (CbR; **b**) rice, and synthetic standard peptide Gln-Asp-Gly-Tyr-Met-Pro-Trp (**c**). Fraction at 27.0 min was collected and further analyzed by LC–TOF/MS while monitoring [M + 2H]^2+^ of *m*/*z* 448.6840 for Gln-Asp-Gly-Tyr-Met-Pro-Trp (**d**–**f**). Arrows indicate MS signals corresponding to RT (21.4 min, upper panel) and *m*/*z* ([M + 2H]^2+^: *m*/*z* 448.6840, lower panel) of standard Gln-Asp-Gly-Tyr-Met-Pro-Trp. AU, arbitrary units; ND, not detected.

Akama et al. recently constructed transgenic CbR to create a foodstuff with antimuscle atrophy properties^21^. Transgenic rice plants were enriched with Cblin peptide by inserting DNA fragments encoding 15 Gln-Asp-Gly-Tyr-Met-Pro-Trp (Cblin-like peptide) repeats processed by chymotrypsin digestion into the rice glutelin gene^21^. Previous studies have shown that four repeats of novokinin hexapeptide and six of lactostatin pentapeptide both with Gln-Arg spacers increased the expression efficacy of functional peptides in rice grain^31,32^. We used Trp as the spacer and avoided basic Arg, which carries a positive charge that reportedly interferes with binding to CBLB^33^. We preserved the backbone structure and inserted Gln and Trp for chymotrypsin recognition and digestion in the stomach to increase the efficiency of expression and release of the inserted peptide sequence, instead of changing the terminal amino acids Asp and Pro, which might result in a loss of function. Akama et al. also confirmed the accumulation of Cblin-like peptides by immunoblotting with an antibody specific to repeats of Gln-Asp-Gly-Tyr-Met-Pro-Trp. We evaluated the digestibility of 15 Cblin peptide repeats in transgenic rice seeds. Glutelin was extracted from CbR or nontransgenic control rice (non-Tg), then digested in vitro with chymotrypsin to identify released Cblin-like peptide using high-performance liquid chromatography (HPLC) and LC–TOF/MS. As digestion progressed, the HPLC peak at RT 26–27 min became intensified in samples derived from CbR (Fig. 4b), but not from non-Tg (Fig. 4a). The RT (27.0 min) of the synthetic peptide standard (Gln-Asp-Gly-Tyr-Met-Pro-Trp) on HPLC (Fig. 4c) matched that of CbR-derived samples (Fig. 4b). To confirm the generation of Cblin-like peptide in the chymotrypsin hydrolysate of CbR, the HPLC fraction at 27.0 min was analyzed by LC–TOF/MS. The RT for standard Cblin-like peptide was 21.4 min and its apparent *m*/*z* was [M + 2H]^2+^ *m*/*z* 448.6840 (Fig. 4f). The MS signal of the 27.0 min HPLC fraction of CbR samples was significant, with a signal-to-noise ratio of 18.0 at 24.1 min in the LC–TOF/MS analysis (Fig. 4e), whereas an MS peak at that RT was undetectable in non-Tg-derived samples (Fig. 4d). This indicated that a peptide with the same *m*/*z* and RT values as Cblin-like peptide occurs in CbR and can be generated in the gut by digestion.

Akama et al. have described the composition of CbR in detail^21^. They found no significant differences in the contents of protein, amino acids, amylose, and starch between non-Tg and CbR. They and others also constructed transgenic rice strains that are rich in other functional peptides using the same technique as that for CbR^34,35^. They showed that the contents of moisture, protein, fat, carbohydrate, fiber, amino acids, fatty acids, minerals (sodium, potassium, iron, calcium, magnesium, and zinc), and vitamins (B1, B2, B6, and niacin) in transgenic rice plants and nontransgenic rice seeds were equivalent. These results suggested that nutritional contents other than overexpressed Gln-Asp-Gly-Tyr-Met-Pro-Trp are essentially identical between non-Tg and CbR and that nonsignificant differences in the nutritional contents are within natural ranges.

### Ability of CbR to prevent muscle atrophy in vivo

**Fig. 5:**
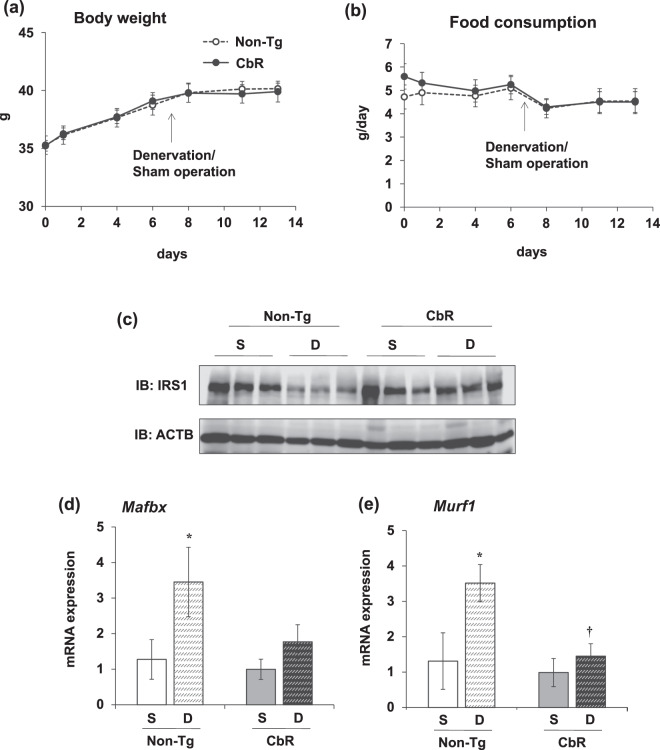
Effects of diets supplemented with Cblin-enriched or non-transgenic rice on sciatic denervated or sham-operated gastrocnemius muscles of mice. Body weight (**a**) and food consumption (**b**) during experimental period in mice fed with diets containing non-transgenic (non-Tg; unfilled circles) or Cblin-supplemented (CbR; filled circles) rice. Results are shown as means ± SEM (*n* = 5–6 per group). Expression of IRS1 protein (**c**) and *Mafbx* (**d**) and *Murf1* (**e**) genes associated with muscle atrophy in sciatic denervated or sham-operated gastrocnemius muscles of mice fed with diets containing non-Tg or CbR. Results are shown as means ± SEM (*n* = 5−6 per group). ^*^P < 0.05 (S vs. D). ^†^*P* < 0.01 (non-Tg vs. CbR). D, denervated; S, sham-operated.

We evaluated the anti-muscle atrophy effects of oral intake of CbR in mice with denervated sciatic nerves. We measured the BW and food consumption of mice fed with rice containing CbR or non-Tg. The BW of mice in both groups increased by 13% during the experimental period with no significant difference (Fig. 5a). The amounts of food consumed by both groups were also similar (Fig. 5b). The wet weight of the gastrocnemius (Ga) and soleus (Sol) muscles decreased by 24% and 45%, respectively, in mice with sciatic denervation that were orally administered with non-Tg (Table 3). We found that CbR significantly suppressed the 13% rate of weight loss caused by denervation in the Ga (Table 3). The intake of CbR suppressed weight loss in the Sol, but this significantly differed between denervated and sham-operated muscle (Table 3). We investigated whether the anti-muscle atrophy effect of CbR correlates with the inhibited degradation of IRS1 protein in Ga muscles of mice fed with CbR. Denervation decreased the total amount of IRS1 protein in mice supplemented with non-Tg. Feeding with CbR restored IRS1 protein even after denervation (Fig. 5c). Along with IRS1 protein preservation, oral CbR intake also suppressed the expression of genes encoding muscle atrophy-related ubiquitin ligases that are regulated by IGF-1 signaling. Expression of the gene encoding the muscle atrophy-related ubiquitin ligase, *Mafbx*, was 2.7-fold higher in denervated Ga muscles of mice fed with non-Tg (Fig. 5d). Meanwhile, *Mafbx* mRNA expression in muscle did not significantly differ between denervated and sham-operated mice fed with CbR (Fig. 5d). Expression of the ubiquitin ligase gene, *Murf1*, was also 2.7-fold higher in mice fed with non-Tg, but significantly suppressed in mice fed with CbR (Fig. 5e). These results indicated that the oral intake of CbR prevented muscle atrophy in mice. Therefore, a standard diet supplemented with CbR might offer an alternative approach to prevent, and perhaps cure muscle atrophy in humans.

**Table 3:** Skeletal muscle weight of mice fed with Cblin peptide-enriched rice.

	Non-Tg		CbR	
	Sham (mg/g BW)	Denervated (mg/g BW)	Sham (mg/g BW)	Denervated (mg/g BW)
Gastrocnemius	166.16 ± 10.27	124.52 ± 5.14*	152.35 ± 11.78	130.23 ± 9.29
Soleus	0.24 ± 0.01	0.13 ± 0.01*	0.22 ± 0.02	0.16 ± 0.02*

BW, body weight; CbR, Cblin-enriched rice; non-Tg, non-transgenic rice.

^*^P < 0.05, sham vs. denervated. Data are expressed as means ± SEM (*n* = 5–6).

We previously found that *Cblb* is expressed at very low levels in normal skeletal muscle. It is upregulated by oxidative stress in skeletal muscle under unloading conditions and is insensitive to insulin/IGF-1^36^. These findings indicated that CbR exerts beneficial effects only on skeletal muscles that are insensitive to insulin/IGF-1 and are accompanied by *Cblb* upregulation. Accordingly, we estimated that CbR cannot increase the sensitivity of normal skeletal muscle to insulin/IGF-1. Due to low *Cblb* expression in skeletal muscle under normal (loaded) circumstances, CbR imposes minimal side effects, because symptoms such as decreased food intake and diarrhea were not found in mice fed with CbR.

We used LC–TOF/MS to analyze CbR-derived peptides in serum and skeletal muscles of mice fed with the transgenic rice. Uptake of the intact Cblin-like heptapeptide, Gln-Asp-Gly-Tyr-Met-Pro-Trp, was undetectable in serum from mice fed with CbR (Supplementary Fig. 2a, b). Although the signals seemed to correspond to the *m*/*z* of the intact Cblin-like heptapeptide in serum from mice fed with CbR, we could not consider that they were from the heptapeptide-derived peak because the RT of 14.5 min did not match that of the synthetic heptapeptide. Akama et al. constructed a CbR containing 15 repeats of Gln-Asp-Gly-Tyr-Met-Pro-Trp^21^ that is released by chymotrypsin digestion (Fig. 4). Chymotrypsin cleaves C-terminal tryptophan, tyrosine, and phenylalanine on peptide chains; thus, Gln-Asp-Gly-Tyr-Met-Pro-Trp, Gln-Asp-Gly-Tyr, or Met-Pro-Trp peptides might be released after oral CbR intake and chymotrypsin digestion. We detected an MS signal at 482.1882 that corresponded to Gln-Asp-Gly-Tyr (Supplementary Fig. 2e). In addition to this peak, several characteristic peaks with diverse *m*/*z* values in serum from mice fed with CbR corresponded to predicted metabolites with ≥4 amino acid residues. The MS signals at 710.2814, 613.2286, 582.2228, and 485.1701 corresponded to Gln-Asp-Gly-Tyr-Met-Pro, Gln-Asp-Gly-Tyr-Met, Asp-Gly-Tyr-Met-Pro, and Asp-Gly-Tyr-Met, respectively (Supplementary Fig. 2c, d, f, g and Supplementary Table 4). The Cblin-like heptapeptide, Gln-Asp-Gly-Tyr-Met-Pro-Trp, released in the stomach might undergo unpredicted modifications, which would generate more peptide fragments during the absorption process. In fact, we detected Gly-Tyr-Met-Pro and Met-Pro in the circulation of rats orally administered with Asp-Gly-Tyr-Met-Pro. However, a CbR-specific signal was not detected in skeletal muscle from mice fed with CbR (Supplementary Fig. 3). Although a signal corresponding to *m*/*z* 596.2537 that is the estimated value of Tyr-Met-Pro-Trp was detected in skeletal muscle of mice fed with CbR, it was not found in all four analyzed mice (Supplementary Fig. 3h) and it was undetectable in serum (Supplementary Fig. 2h). Based on these findings, we speculated that orally administered CbR is digested in the gut, then characteristic shorter peptides released into the circulation elicit bioactivities that restore muscle mass. The lack and/or the difficulty of reproducibility in detection might be due to low content of the heptapeptide and its metabolites in skeletal muscle. Intra- and interindividual differences such as age^18^, the time of day^37^, and the duration of feeding can alter the absorption and distribution of peptides after oral intake. Muscle peptide accumulation after oral intake has not been reported. Therefore, amounts of muscle peptide uptake and the involved mechanism require further evaluation. We plan to detect the peptides in skeletal muscle considering these factors and elucidate the absorption and muscle accumulation of this peptide, its metabolites, and the individual bioactivities of identified metabolite fragments.

As described above, CbR-derived Gln-Asp-Gly-Tyr-Met-Pro-Trp and its metabolic fragments containing the backbone structure seems to be responsible for the functionality of CbR. The amino acid sequence of Cblin pentapeptide was designed to inhibit interaction between CBLB and IRS1 proteins based on the ability of CBLB to recognize and bind to phosphorylated tyrosine^38^. Metabolites containing tyrosine might also inhibit CBLB binding to IRS1 via their tyrosine residues even when they are not phosphorylated. We previously elucidated the crystal structure of the TKB domain of CBLB complexed with Cblin and found that Tyr in Cblin is essential to conserve hydrophobic interaction between CBLB and Cblin^9^. In accordance with this, we confirmed the suppressive effect of the shortened Cblin pentapeptide, Gly-pTyr-Met, on CBLB-mediated ubiquitination of IRS1 in vitro^9^. Furthermore, the modified Cblin peptide Asp-Ile-Tyr-Asn-Pro containing intact (non-phosphorylated) tyrosine also suppresses CBLB-mediated IRS1 ubiquitination^19^. We did not detect all metabolites in mice given CbR, but those of Cblin-like peptide containing Tyr might have synergistically functioned with intact peptides to inhibit CBLB activity and restore muscle mass. We have scant understanding of which fragments are generated, and how they contribute to the functionality of CbR, but we plan to investigate the absorption behavior and bioactivity of Gln-Asp-Gly-Tyr-Met-Pro-Trp, and screen possible peptide fragments generated by dietary CbR and the relative contribution of each to CbR functionality. These complex topics will be time-consuming, but we will elucidate them.

We estimate that trypsin, which prefers peptide linkages where the carboxylic acid group is provided by a basic amino acid (arginine, lysine, and histidine), does not digest Cblin-like peptide. On the other hand, pepsin has specific preferences, as it selectively targets bonds containing the amine group of aromatic amino acids (tryptophan, tyrosine, and phenylalanine). CbR-derived Cblin-like peptides might be digested as: Gln-Asp-Gly ↓ Tyr-Met-Pro ↓ Trp. However, pepsin is responsible for digesting only ~15% of dietary protein^39^. Upon CbR feeding, both pepsin and chymotrypsin would function to release Cblin-like peptide and its metabolites, but more peptides might be generated by chymotrypsin than pepsin.

The sequence encoding repeated-Cblin-like peptides derived from CbR is inserted into that encoding glutelin, the major storage protein in rice. Glutelin is stored within protein body (PB)-II in the rice endosperm, while another storage protein, prolamine, is stored in PB-I^40^. Whereas PB-II is easily digested in humans by proteolytic enzymes, PB-I is resistant to digestion by gastrointestinal enzymes such as pepsin and is excreted^41^. Although we could not exclude the possibility that some nutrients in CbR affect glutelin digestibility, the deposition sites of inserted peptides are considered important to determine the efficiency of glutelin digestion and to acquire the beneficial effect of the inserted peptides. Takaiwa et al. developed transgenic rice seeds overexpressing cedar pollen allergen and found that the tolerogenic dose required to achieve allergen-specific immune tolerance by oral feeding differs between transgenic seeds that use PB-I or PB-II as deposition sites^42^. We estimate that PB-II sites containing glutelin with Cblin-like peptides are easily digested in the mouse GI tract and release these peptides, but future studies are needed to determine the digestion and absorption mechanism in vivo.

Coexisting components in food matrices might affect di-/tripeptide absorption by inhibiting or facilitating PEPT1 function^43^. For example, egg-white protein hydrolysate contains oligopeptides that inhibit peptidase-mediated epithelial hydrolysis, thus contributing to the absorption of longer peptides^44^. The metabolic behavior and functionality of Gln-Asp-Gly-Tyr-Met-Pro-Trp and its fragment peptides upon CbR intake remained to be determined. Here, CbR digestion in vitro seemed to generate several peptide fragments (Fig. 4). How these fragments and other protein/non-protein components of rice affect the digestion and absorption of Cblin peptide remains unknown. Exosomes or exosome-like vesicles might be involved in the intestinal uptake and systemic delivery of Cblin peptides. Further study is needed to uncover the absorption mechanism of Cblin peptides in the CbR food matrix.

The present study showed that intact Cblin peptide can circulate in the blood of rodents by passing from epithelial cells via tight junctions. Cblin peptide might synergistically function with its metabolites to suppress muscle atrophy in rodents. A standard diet supplemented with CbR could become an alternative approach to preventing, and perhaps even curing, muscle atrophy in humans.

## Methods

### Transport of Cblin pentapeptides across Caco-2 cell monolayers

Cell culture and transport experiment were performed as described^24,45^. Caco-2 cells were cultured using BD BioCoat Intestinal Epithelium Differentiation Environment kits (BD Bioscience, Franklin Lakes, NJ, USA). Caco-2 cells grown in 0.9 cm^2^ Falcon cell culture inserts (polyethylene terephthalate membranes; pore size, 1.0 μm; Becton Dickinson and Co.) with a type I collagen-coated membrane (Cellmatrix type IA Collagen Gel Culturing Kit; Nitta Gelatin Inc., Osaka, Japan) were gently rinsed twice with Hanks balanced salt solution (HBSS; 137 mM NaCl, 5 mM KCl, 5.5 mM D-glucose, 4 mM NaHCO_3_, 0.75 mM NaHPO_4_·12H_2_O, 0.4 mM KH_2_PO_4_, 0.8 mM MgSO_4_·7H_2_O, and 1.26 mM CaCl_2_·2H_2_O, pH 6.0 adjusted with 10 mM 2-(N-morpholino)-ethanesulfonic acid). A portion (1.5 mL) of HBSS buffer (pH 6.0) was added to the apical side, and HBSS buffer (pH 7.4 adjusted with 10 mM *N*-2-hydroxyethylpiperazine-*N*′-2-ethanesulfonic acid) was added to the basolateral side. After equilibration for 15 min at 37 °C, the apical buffer was replaced with fresh HBSS (pH 6.0) containing 1 mM peptide and 0.5% DMSO. Portions (60 μL) of solutions were drawn from the apical or basolateral sides at 15, 30, 45, and 60 min to determine amounts of transported peptides. Monolayer integrity was evaluated by measuring TEER using a Millicell^®^ ERS-2 Voltohmmeter (Merck KGaA, Darmstadt, Germany), and monolayers with a TEER of >300 Ω · cm^2^ were included in the experiments. The apparent permeability coefficient (*P*_app_, cm/s) was calculated as

1 $${\it{P}}_{{{{\mathrm{app}}}}}\left( {{{{\mathrm{cm/}}}}{\mathrm{s}}} \right) = {\it{V/AC}}_{\it{0}} \times {{{\mathrm{d}}}}{\it{C}}{{{\mathrm{/d}}}}{\it{t}}$$

where d*C*/d*t* is the change in concentration on the basolateral side over time (mM/*s*), *V* is the volume of solution in the basolateral compartment (1.5 mL), *A* is the surface area of the membrane (0.9 cm^2^), and *C*_*0*_ is the initial concentration on the apical side (mM).

We investigated the involvement of PEPT1 in peptide transport by competition studies using 10 mM Gly-Sar, a PEPT1 substrate^46^. We also investigated peptide transport over paracellular tight junctions by incubating monolayers with the tight junction opener, cytochalasin D (0.5 μg/mL) for 30 min^47^ before evaluating transport.

### Quantitation of transported oligopeptides

Oligopeptides were separated at 40 °C using a linear gradient of 0.1% formic acid (FA) in MS grade water (solvent A) and 0.1% FA in acetonitrile (ACN) (solvent B) for 20 min at a flow rate of 0.2 mL/min, and an Agilent 1200 series HPLC system (Agilent Technologies Inc., Santa Clara, CA, USA) equipped with a Cosmosil 5C_18_-MS-II column (Φ 2.0 mm × 150 mm, Nacalai Tesque Inc., Kyoto, Japan). The MS conditions comprised: drying gas, N_2_; flow rate, 8.0 L/min; drying gas temperature, 200 °C; drying gas pressure, 1.6 bar; HV capillary voltage, −4500 V; capillary exit, 70.0 V; skimmer 1, 50.0 V; hexapole 1, 23.0 V; hexapole RF, 100.0 Vpp; skimmer 2, 23.0 V; lens 1 transfer, 52.0 μs, and mass range *m*/*z* 100–1000. A calibration solution of 10 mM sodium formate in 50% ACN was injected at the beginning of each run. Data were analyzed using Bruker Data Analysis version 3.2 software (Bruker, Billerica, MA, USA). Calibration curves were prepared using 0.1–10 μM standards.

### Detection of orally administered oligopeptides in rats

Seven-week-old male SD (Crj:SD) rats (Charles River Japan, Kanagawa, Japan) were housed for 1 week at 21 ± 1 °C with lights on from 8 a.m. to 8 p.m. and given a standard MF chow diet (Oriental Yeast Co., Tokyo, Japan) with access to water *ad libitum*. The rats were fasted for 16 h before being orally administered once with Asp-Gly-Tyr-Met-Pro (100 mg/kg BW) dissolved in milli-Q water. Blood samples from the tail veins of the rat at 0, 15, 30, 45, and 60 min thereafter were placed in chilled microtubes containing ethylenediaminetetraacetic acid disodium salt (EDTA-2Na, 0.2 mg) and protease inhibitors (aprotinin, 0.1 mg; chymostatin, 0.1 mg), then plasma was obtained by immediate centrifugation at 3500 × *g* for 15 min. The plasma samples (100 μL) were spiked with Asp-Gly-Tyr-Met-Pro labeled with stable isotopes (Asp-Gly[^13^C_2_; ^15^N]-Tyr-Met-Pro) at a final concentration of 50 pmol/mL. Plasma was precipitated with ice cold ACN containing 0.1% FA, separated by centrifugation at 14,000 × *g* for 15 min, then the supernatant was collected and dried using a centrifugal evaporator. Pellets were resuspended in 25 μL of 0.1% FA and analyzed under the LC–TOF/MS conditions described above. Plasma concentrations of Asp-Gly-Tyr-Met-Pro were calculated using the internal standard method (Eq. (2)) where *y* is the ratio between the observed peak area of Asp-Gly-Tyr-Met-Pro and the internal standard Asp-Gly(^13^C_2_; ^15^N)-Tyr-Met-Pro, and *x* is the peptide concentration (pmol/mL)

2 $${{{\mathrm{Asp - Gly - Tyr - Met - Pro,}}}}\;{\it{y}} = 0.0464{\it{x}} - 0.0245(0 - 100\;{{{\mathrm{pmol/mL,}}}}\;{{{{r}}}} = 0.9982)$$

### Detection of Cblin-like peptides released from Cblin-enriched transgenic rice seeds after chymotrypsin digestion in vitro

Akama et al. have constructed transgenic Cblin peptide-enriched rice (CbR)^21^. Rice flour (1 g) prepared from CbR or non-transgenic rice (non-Tg) was delipidized with acetone (10 mL) and dried at ambient temperature for 24 h. Glutelin was then extracted from the rice flour twice with 4 mL of 0.02 M NaOH (pH 12.0) at 20 °C for 30 min and separated by centrifugation at 3000 × *g* for 30 min. Glutelin precipitated from combined supernatants by adjusting the isoelectric point to pH 5.0 was washed twice with distilled water and dissolved in 100 mM Tris-HCl (pH 7.8) containing 10 mM CaCl_2_. Glutelin protein content was measured using Pierce BCA Protein Assay Kits (Thermo Fisher Scientific Inc., Waltham, MA, USA). Glutelin (1 mg) was digested in vitro with chymotrypsin (Sigma Aldrich Corp., St. Louis, MO, USA) at a ratio (w/w) of 1:60 at 30 °C for 120 min. Thereafter, protease inhibitor tablets (Sigma Aldrich Co.) were added, then samples were concentrated by vacuum evaporation and dissolved in milli-Q water containing 0.1% trifluoroacetic acid (TFA) for HPLC analysis.

Samples were separated using a Hitachi 5110 HPLC system connected to a Chromaster Organizer, Interface Box, 5430 Diode Array Detector, and 5110 Pump (Hitachi Ltd., Tokyo, Japan) with a TSKgel ODS-80Ts column (2.0 mm × 25 cm, 5 μm; Tosoh Co., Kanagawa, Japan), with a gradient elution of 0.1% TFA (solvent A) in ultrapure water to 0.1% TFA in ACN (solvent B) over 60 min at a flow rate of 0.2 mL/min: 0–5 min, 5% B isocratic; 5–30 min, 5–60% B; 30–40 min, 60–70% B; 41–45 min, 70–100% B; 45–50 min, 100% B isocratic; 50–55 min, 100–5% B; 55–60 min, 5% B isocratic. Chromatograms were obtained at 220 nm. Data signals were processed using a Chromaster System Manager (Hitachi Ltd.). The fraction at RT 27.0 min (corresponding to synthetic Gln-Asp-Gly-Tyr-Met-Pro-Trp) was further analyzed by LC–TOF/MS as described above except for the capillary exit and hexapole RF being 60 V and 100 Vpp, respectively.

### Determination of effects of transgenic Cblin peptide-enriched rice on mice with sciatic denervation

Six-week-old male Jcl:ICR mice (Japan SLC Inc., Shizuoka, Japan) were housed with access to a standard diet and water *ad libitum* under lights on from 8 a.m. to 8.0 p.m. The experimental diet comprised powdered Cblin rice grains (CbR) and non-transgenic rice (non-Tg) mixed with the standard diet (50% w/w). Supplementary Table 2 shows the composition of the diets. The mice were then fed with the diet containing either CbR or non-Tg for 7 days, then their sciatic nerves were unilaterally transected under anesthesia. The contralateral innervated (sham-operated) muscles of the same animals served as controls. Seven days later, the mice were sacrificed and the Sol and Ga muscles were dissected, weighed, and frozen in liquid nitrogen.

All animal experiments proceeded according to the guidelines for animal experiments at Tokushima University and Kyushu University. The Ethics Review Committee for Animal Experimentation at these institutions approved all the experimental protocols described herein (Permission Nos. T27–113 and A30-015-4).

### Real-time reverse transcription-polymerase chain reaction (RT-PCR)

Total RNA was extracted using ISOGEN (Nippon Gene, Osaka, Japan). Single-stranded cDNA was synthesized using M-MLV Reverse Transcriptase (Promega). Real-time RT-PCR proceeded using SYBR-Green Master Mix (Thermo Fisher Scientific Inc., Waltham, MA, USA) and a Real-time PCR system (Thermo Fisher Scientific Inc.). Supplementary Table 3 shows the primer sequences. Amounts of target mRNA were normalized relative to that of *Actb*.

### Immunoblotting

Proteins were extracted from mouse muscles into Tris-HCl buffer, pH 7.5, containing 150 mM NaCl, 5 mM EDTA, 1% TritonX-100, 10 mM NaF, 2 mM Na_3_VO_4_, 10 μM MG132, and protease inhibitor tablets (Roche Diagnostics, Basel, Switzerland). Total protein extracts were resolved by SDS-PAGE and transferred to PVDF membranes. PBS-Tween containing 4% Block Ace Powder (DS Pharma Biomedical Co. Ltd., Osaka, Japan) was used to block non-specific binding. Then, the membranes were incubated with primary antibodies at 4 °C overnight. Chemiluminescent blot was detected using ImmunoStar LD (Wako Pure Chemical Industries, Osaka, Japan) and signals were quantified by densitometry. All blots or gels were derived from the same experiment and were processed in parallel. The primary antibodies were anti-IRS1 (Merck Millipore Burlington, MA, USA) and anti-ACTIN (ABclonal Technology, Woburn, MA, USA).

### Statistical analysis

Values were statistically evaluated by Student’s *t* tests using Excel-Toukei 2015 software (Social Survey Research Information Co. Ltd., Osaka, Japan), and are expressed as means ± SEM. Statistical significance among *P*_app_ of Asp-Gly-pTyr-Met-Pro, Asp-Gly-Tyr-Met-Pro, and Gly-Sar-Sar-Sar-Sar was assessed by one-way analyses of variance (ANOVA) followed by Tukey *post hoc* tests. Data from mice fed with non-Tg or CbR were statistically evaluated by two-way ANOVA followed by Tukey–Kramer multiple comparisons for *post hoc* analysis using Excel-Toukei 2015 software. Values with *P* < 0.05 were considered significantly different.

### Reporting summary

Further information on research design is available in the Nature Research Reporting Summary linked to this article.

## Supplementary information

Supplementary Information

Reporting Summary

## Data Availability

The authors declare that all data supporting the findings of this study are available within the paper and supplementary information.
