# Supplementary material for: Oral intake of rice overexpressing ubiquitin ligase inhibitory pentapeptide prevents atrophy in denervated skeletal muscle
Source: NPJ Sci Food. 2021 Sep 9;5:25. doi: 10.1038/s41538-021-00108-0 (PMC8429733; doi:10.1038/s41538-021-00108-0)
Supplement: Supplementary file 1 — Supplementary Information [file 41538_2021_108_MOESM1_ESM.pdf]

Oral intake of rice overexpressing ubiquitin ligase inhibitory pentapeptide prevents atrophy in denervated skeletal muscle

Reiko Nakao[1*], Weilin Shen[2*], Yasuka Shimajiri[3, 4*], Kumiko Kainou[3], Yuki Sato[1], Anayt Ulla[1], Kohta Ohnishi[5], Miyuki Ninomiya[1], Ayako Ohno[1], Takayuki Uchida[1], Mitsuru Tanaka[2], Kazuhito Akama[3], Toshiro Matsui[2], Takeshi Nikawa[1]

[1]Department of Nutritional Physiology, [5]Department of Clinical Nutrition and Food Management, Institute of Biomedical Sciences, Tokushima University Graduate School, Tokushima, Tokushima 770-8503, Japan

[2]Division of Bioresources and Bioenvironmental Sciences, Faculty of Agriculture, Graduate School, Kyushu University, Fukuoka, Fukuoka 819-0395, Japan

[3]Department of Life Science, Shimane University, Matsue, Shimane 690-8504, Japan

[4]EditForce, Fukuoka, Fukuoka 810-0001, Japan

[*]These authors contributed equally to this work.

Supplementary information

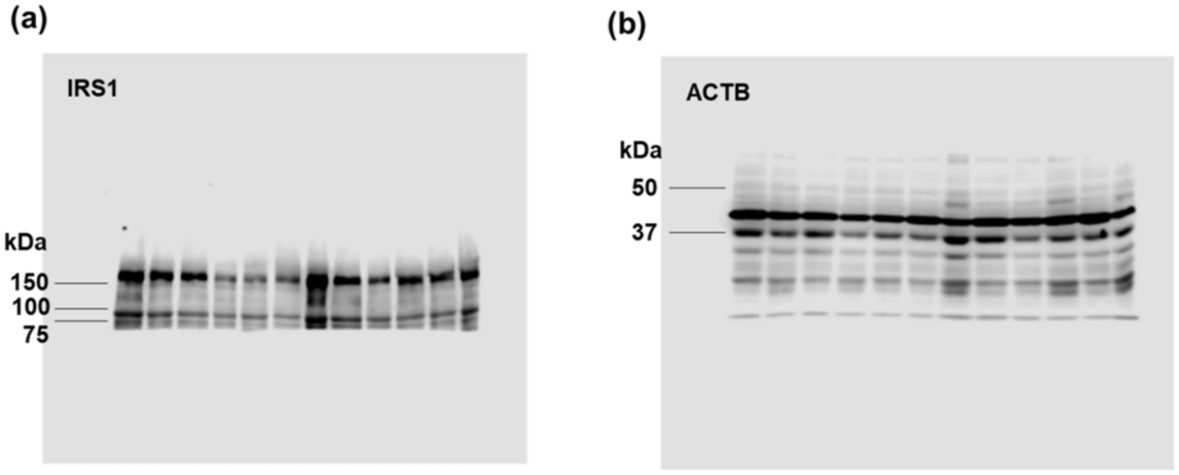

Supplementary Figure 1. Full-size blots of polyacrylamide gels.

Images of actual IRS1 and ACTB blots shown in Figure 5. Image size varies because membrane was cut to blot several proteins.

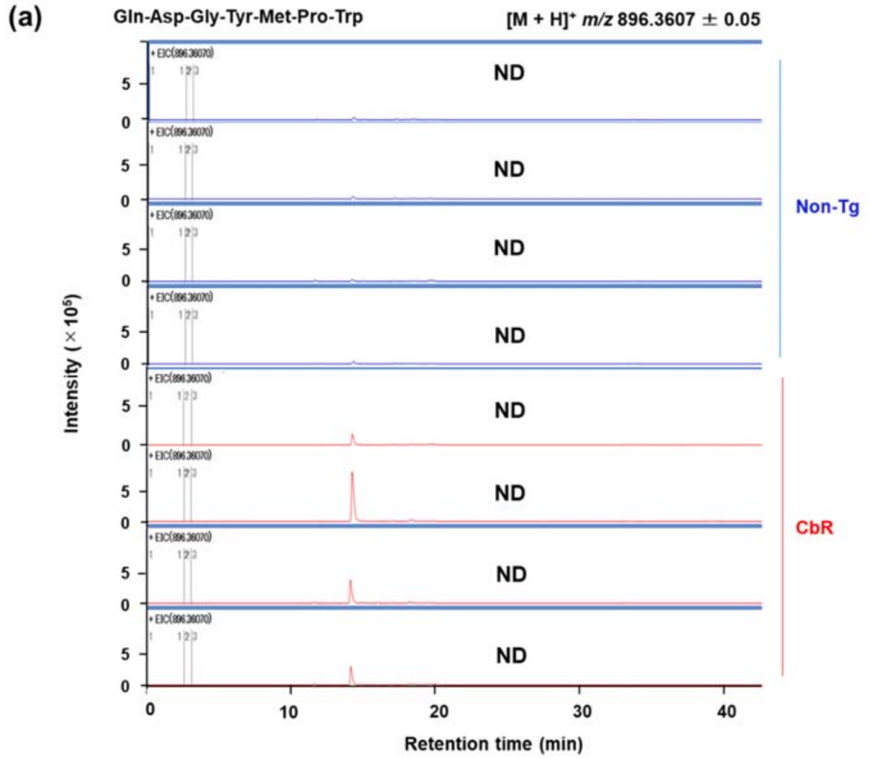
(a)
Gln-Asp-Gly-Tyr-Met-Pro-Trp
[M + H]+ m/z 896.3607 ± 0.05
ND
Non-Tg
CbR
Intensity (×10^5)
Retention time (min)

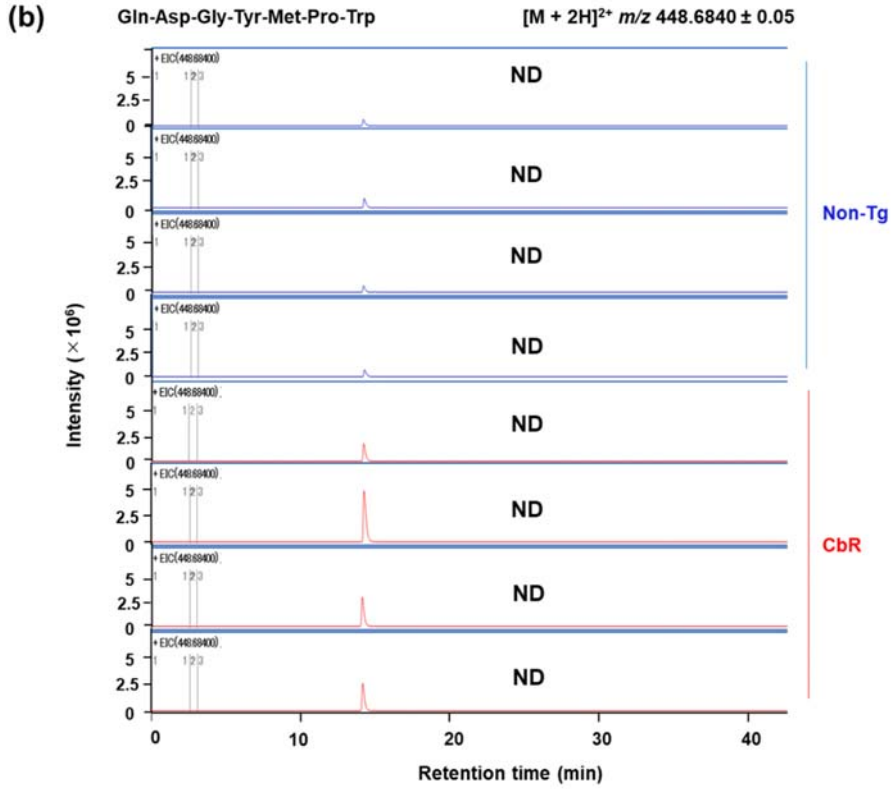
(b)
Gln-Asp-Gly-Tyr-Met-Pro-Trp
[M + 2H]2+ m/z 448.6840 ± 0.05
ND
Non-Tg
CbR
Intensity (×10^6)
Retention time (min)

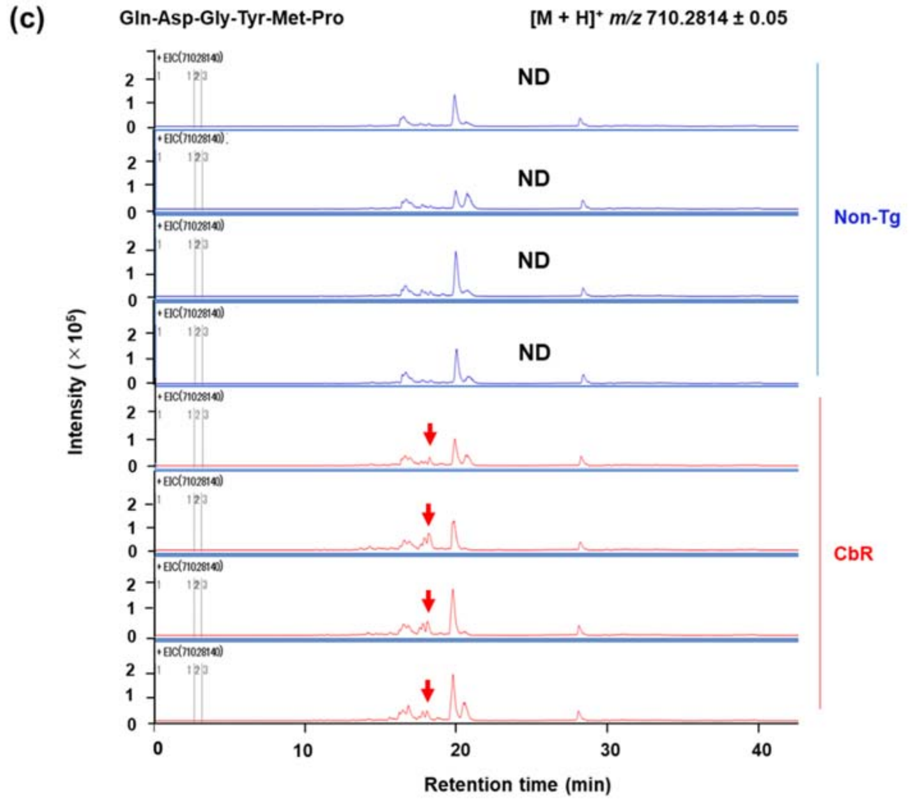
(c)
Gln-Asp-Gly-Tyr-Met-Pro
[M + H]+ m/z 710.2814 ± 0.05
+EIC(710.28140)
ND
Non-Tg
CbR
Intensity (×10⁵)
Retention time (min)

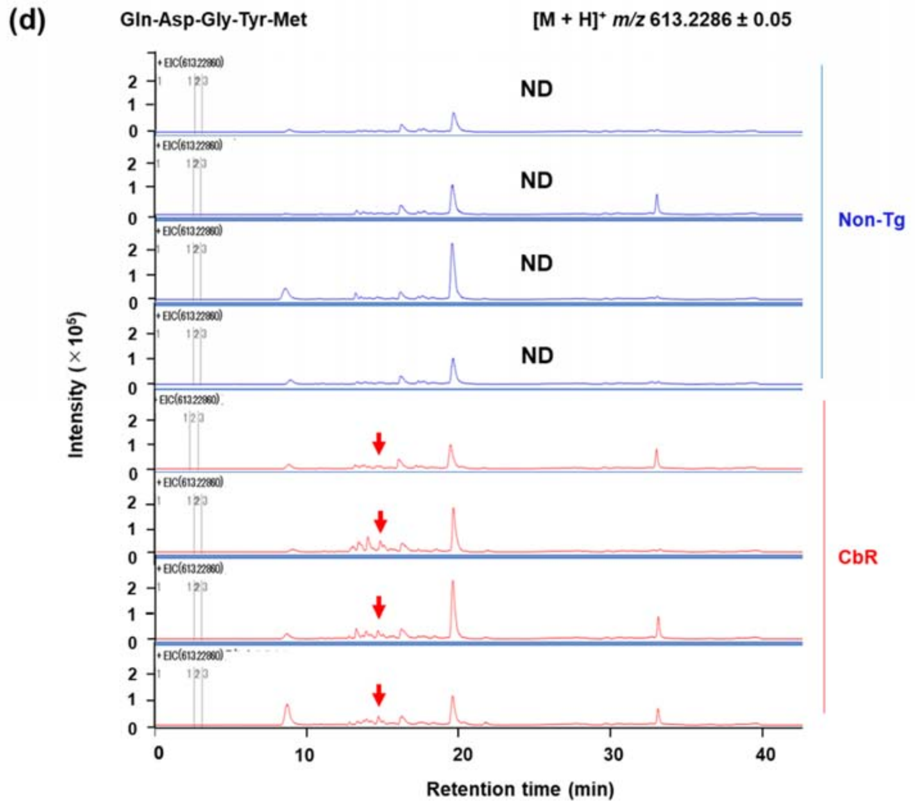
(d)
Gln-Asp-Gly-Tyr-Met
[M + H]+ m/z 613.2286 ± 0.05
+EIC(613.22860)
ND
Non-Tg
CbR
Intensity (×10⁵)
Retention time (min)

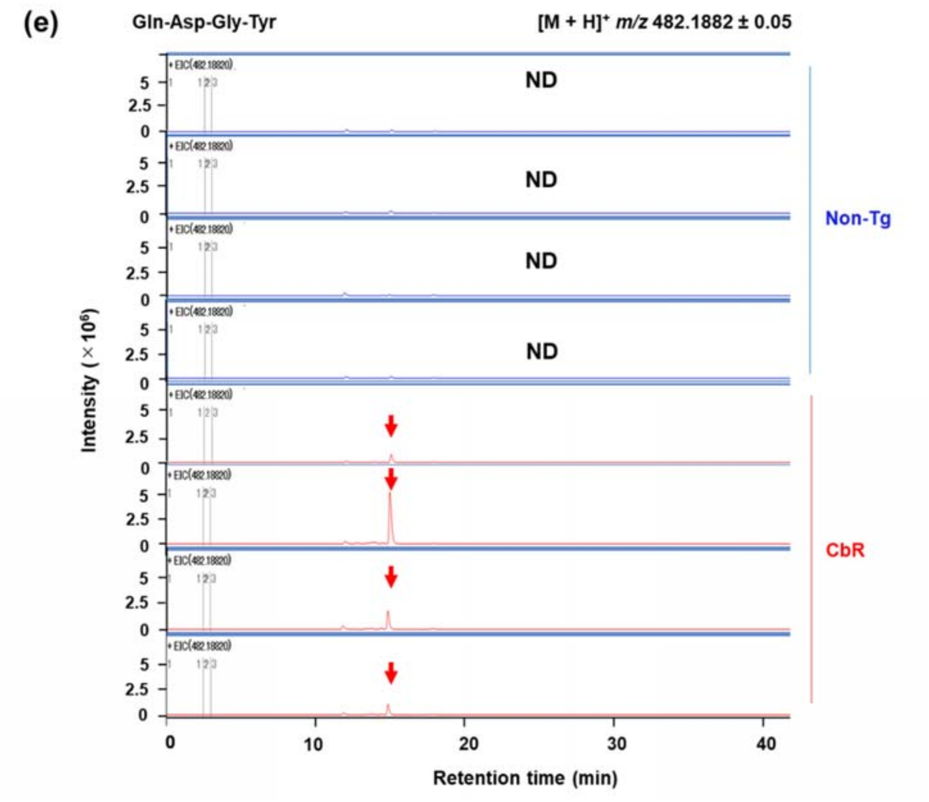
(e)
Gln-Asp-Gly-Tyr
[M + H]+ m/z 482.1882 ± 0.05
ND
ND
ND
ND
Non-Tg
CbR
Intensity (×10^6)
Retention time (min)

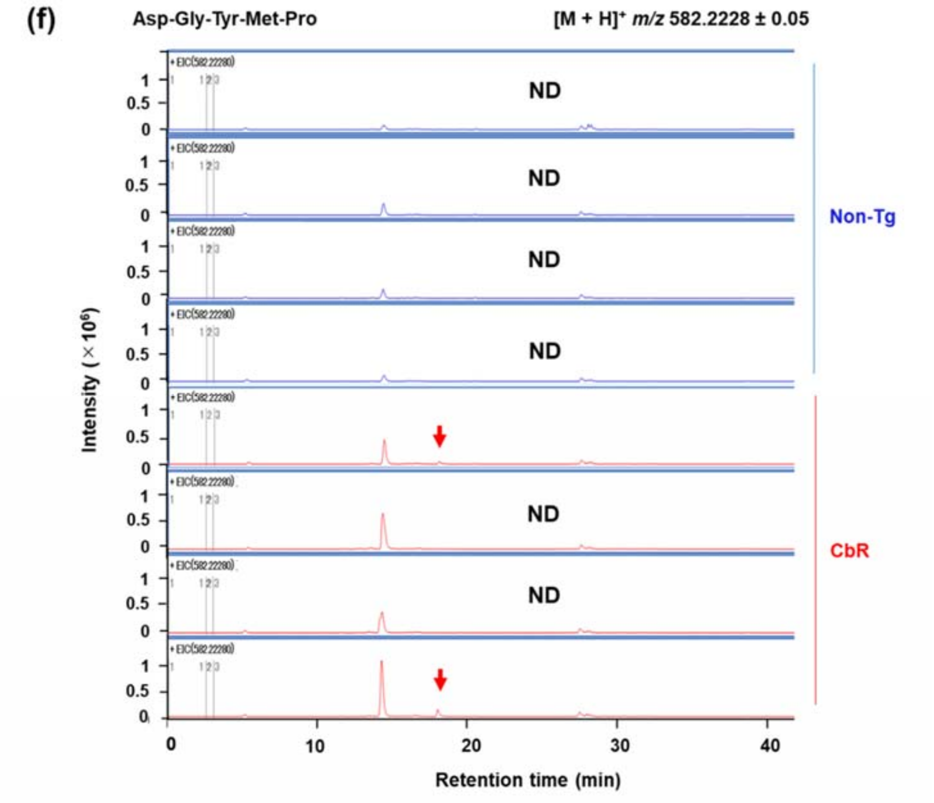
(f)
Asp-Gly-Tyr-Met-Pro
[M + H]+ m/z 582.2228 ± 0.05
ND
ND
ND
ND
ND
ND
Non-Tg
CbR
Intensity (×10^6)
Retention time (min)

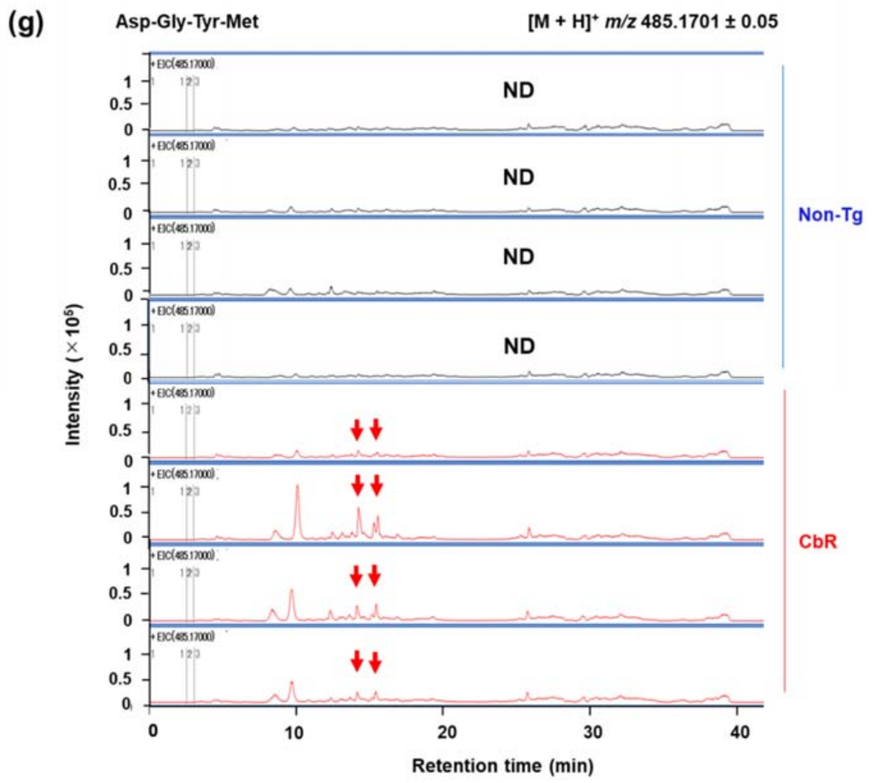
(g)
Asp-Gly-Tyr-Met
[M + H]+ m/z 485.1701 ± 0.05
ND
Non-Tg
CbR
Intensity (×10⁵)
Retention time (min)

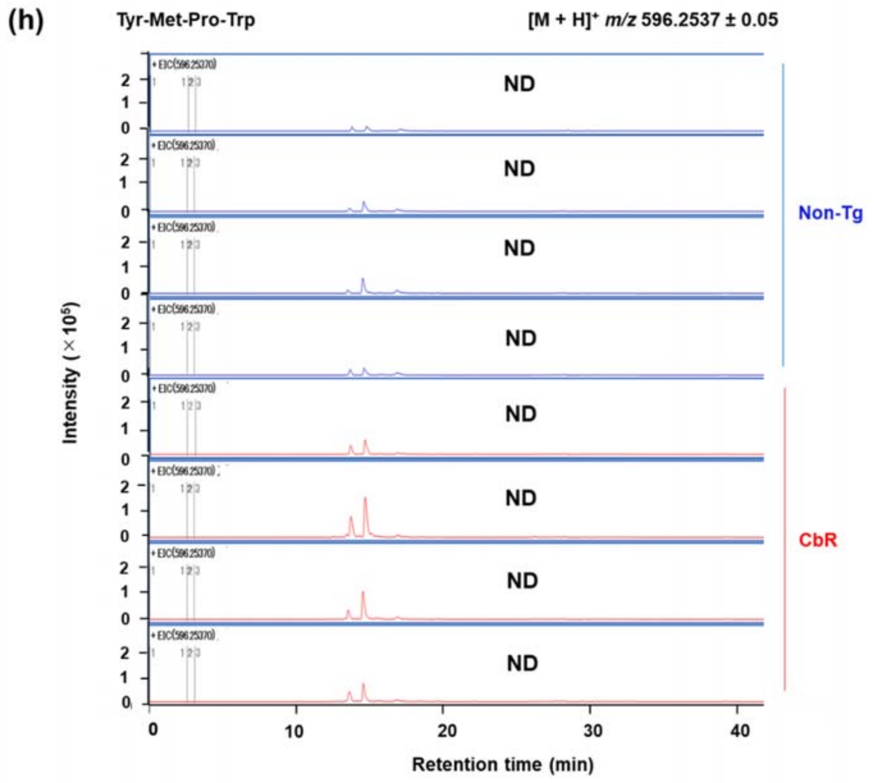
(h)
Tyr-Met-Pro-Trp
[M + H]+ m/z 596.2537 ± 0.05
ND
Non-Tg
CbR
Intensity (×10⁵)
Retention time (min)

Supplementary Figure 2. Analysis of serum collected from mice fed with Non-Tg or CbR using LC-TOF/MS.

Mice were handled as described in Methods. Briefly, 6-week-old male Jcl:ICR mice (Japan SLC Inc., Shizuoka, Japan) were housed with access to a standard diet and water *ad libitum*. The mice were then fed with a diet containing either CbR or Non-Tg for 7 days, then their sciatic nerves were unilaterally transected under anesthesia. Seven days later, serum was separated from blood by centrifugation for 10 min at 5,800 × g, then stored at -80 °C. Serum was deproteinized by precipitation with ice cold ACN containing 0.1% FA then centrifuged at 14,000 × *g* for 15 min. The supernatant was collected and dried using a centrifugal evaporator, and pellets were resuspended in 50 μL of 0.1% FA and analyzed by LC-TOF/MS under conditions described in Methods. Extracted ions correspond to those of possible metabolites. Arrows indicate MS signals specific for CbR group. ND, not detected.

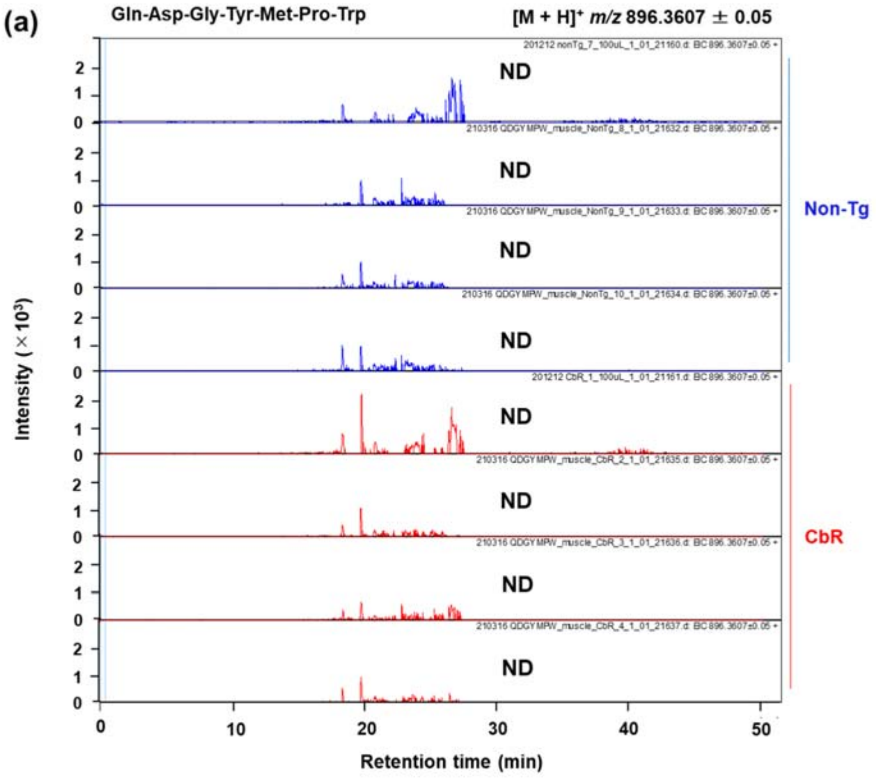
(a)
Gln-Asp-Gly-Tyr-Met-Pro-Trp
[M + H]+ m/z 896.3607 ± 0.05
ND
Non-Tg
CbR
Intensity (×10³)
Retention time (min)
0
10
20
30
40
50

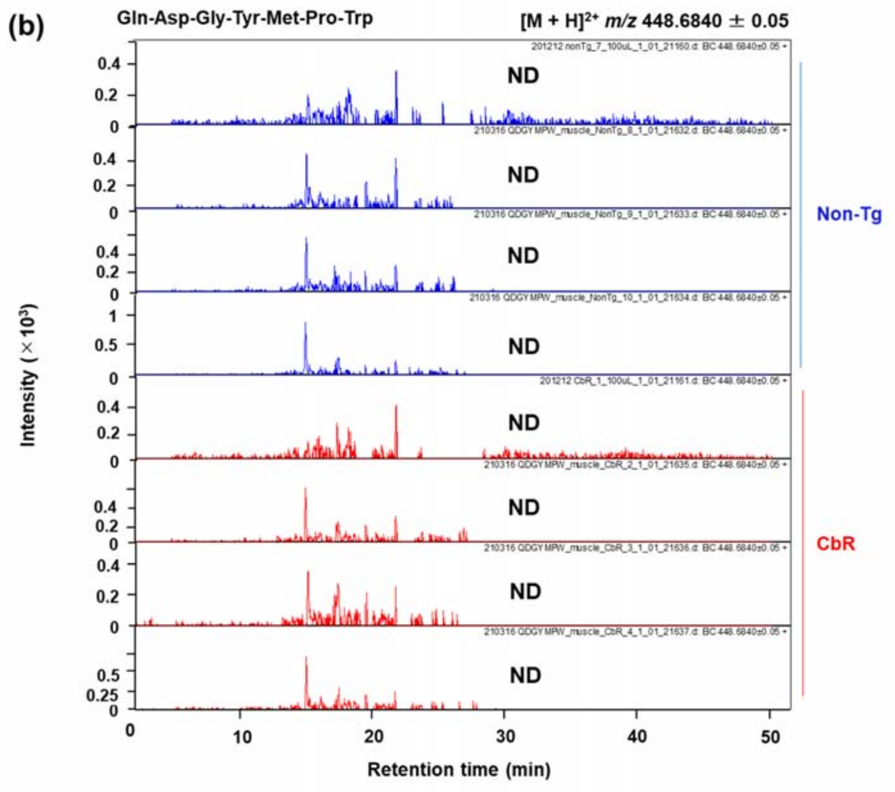
(b)
Gln-Asp-Gly-Tyr-Met-Pro-Trp
[M + H]2+ m/z 448.6840 ± 0.05
ND
Non-Tg
CbR
Intensity (×10³)
Retention time (min)
0
10
20
30
40
50

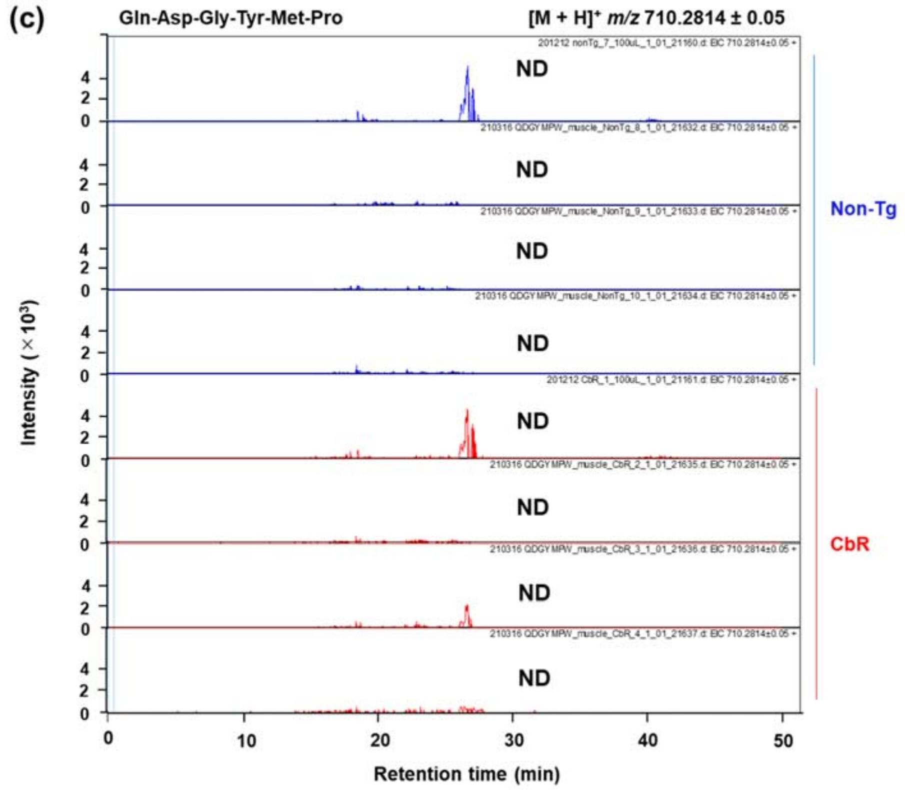

(c)
Gln-Asp-Gly-Tyr-Met-Pro
[M + H]+ m/z 710.2814 ± 0.05
ND
Non-Tg
CbR
Intensity (×10³)
Retention time (min)
0
10
20
30
40
50

46

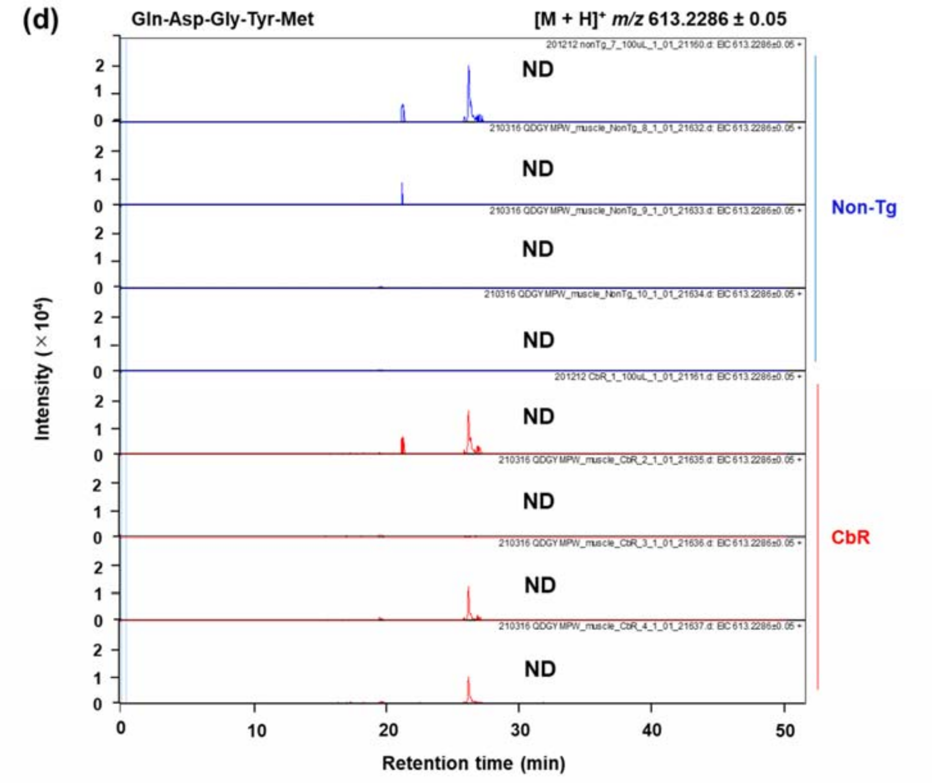

(d)
Gln-Asp-Gly-Tyr-Met
[M + H]+ m/z 613.2286 ± 0.05
ND
Non-Tg
CbR
Intensity (×10⁴)
Retention time (min)
0
10
20
30
40
50

47

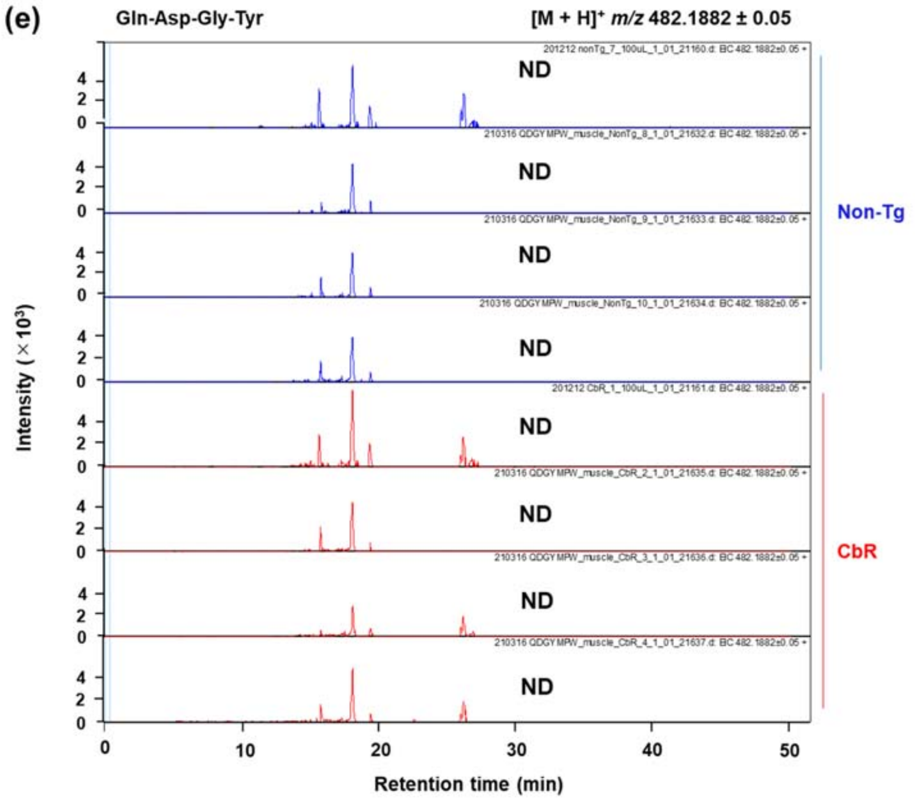
(e)
Gln-Asp-Gly-Tyr
[M + H]+ m/z 482.1882 ± 0.05
ND
Non-Tg
CbR
Intensity (×10³)
Retention time (min)
0
10
20
30
40
50

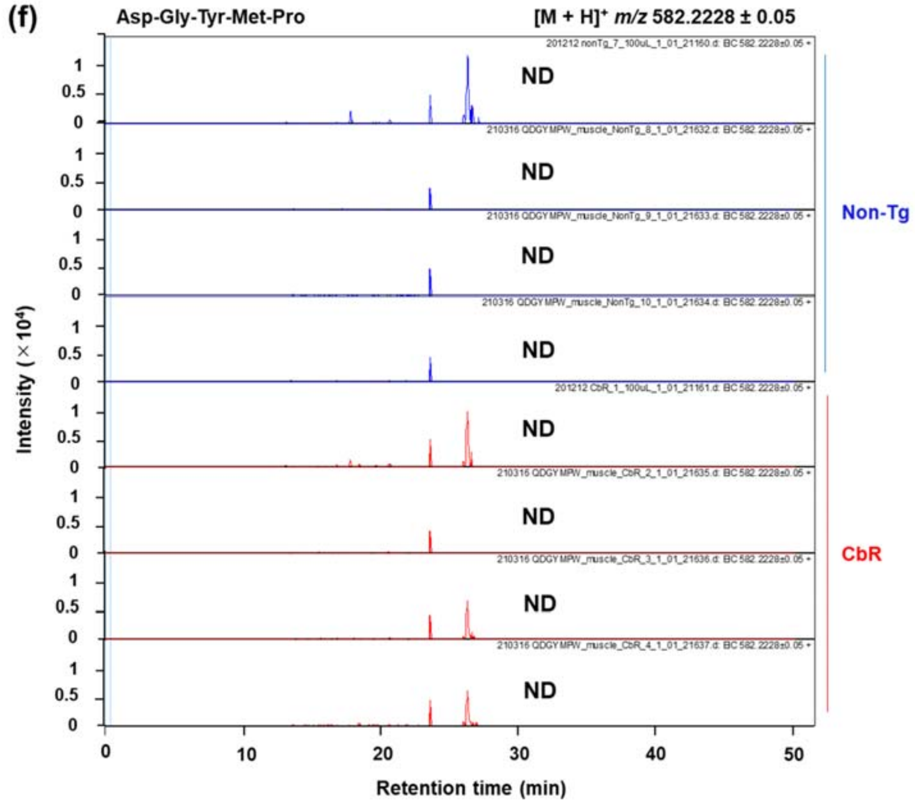
(f)
Asp-Gly-Tyr-Met-Pro
[M + H]+ m/z 582.2228 ± 0.05
ND
Non-Tg
CbR
Intensity (×10⁴)
Retention time (min)
0
10
20
30
40
50

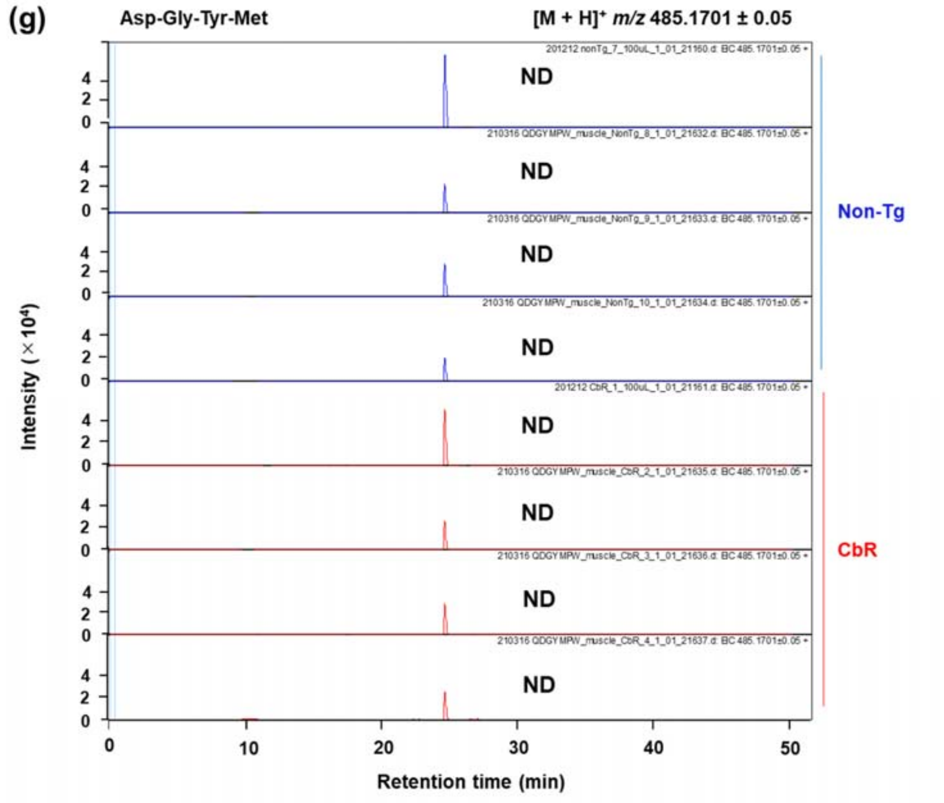

(g)
Asp-Gly-Tyr-Met
[M + H]+ m/z 485.1701 ± 0.05
ND
Non-Tg
CbR
Intensity (×10⁴)
Retention time (min)

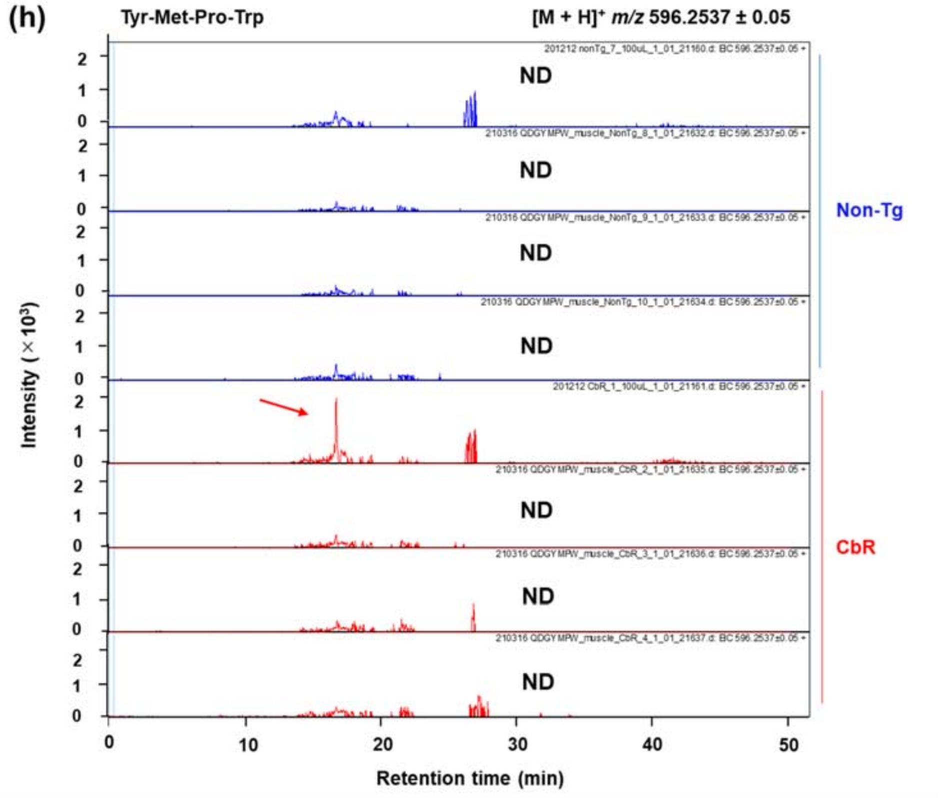

(h)
Tyr-Met-Pro-Trp
[M + H]+ m/z 596.2537 ± 0.05
ND
Non-Tg
CbR
Intensity (×10³)
Retention time (min)

Supplementary Figure 3. LC-TOF/MS analysis of skeletal muscle collected from mice fed with Non-Tg or CbR.

Mice were handled as shown in Methods and in Supplementary Fig. 2. Gastrocnemius (Ga) muscles stored at -80°C were homogenized in Tris-HCl buffer, pH 7.5, containing 150 mM NaCl, 5 mM EDTA, 1% TritonX-100, 10 mM NaF, 2 mM Na_3VO_4, 10 μM MG132, and protease inhibitor tablets (Roche Diagnostics). Lysates were precipitated with ice cold ACN containing 0.1% FA, then separated by centrifugation at 14,000 × *g* for 15 min. Supernatants were collected and dried using a centrifugal evaporator. Pellets were resuspended in 100 μL of 0.1% FA and analyzed by LC-TOF/MS under conditions described in Methods. Extracted ions correspond to those of possible metabolites. Arrows indicate MS signals specific to CbR. ND, not detected.

Supplementary Table 1. Pharmacokinetics after a single oral administration of Asp-Gly-Tyr-Met-Pro at lower or higher doses in 8-wk SD rats.

Measured parameters	Oral peptide dose (mg/kg BW)	
	50	200
C_{max} (pmol/mL plasma)	0.79	27.25
t_{max} (min)	10	30
$AUC_{0-60\ min}$ (pmol · min/mL plasma)	14.61	648.91
$t_{1/2}$ (min)	8.99	5.58

Data are expressed as means (n = 1 per dose). $AUC_{0-60\ min}$, area under plasma concentration vs. time curve; BW, body weight; C_{max}, maximum plasma concentration; t_{max}, elapsed time to peak plasma concentration; $t_{1/2}$, elimination half-life.

Supplementary Table 2. Composition of diets.

	Standard diet for acclimation	Non-Tg	CbR
Non-transgenic rice (g)	-	50.0	-
Cblin peptide-enriched rice (g)	-	-	50.0
Casein (g)	19.8	16.6	16.6
Corn starch (g)	46.2	-	-
Lard (g)	4.4	3.5	3.5
Sucrose (g)	23.1	23.4	23.4
Cellulose (g)	2.0	2.0	2.0
AIN93 vitamin mix (g)	1.0	1.0	1.0
AIN93 mineral mix (g)	3.5	3.5	3.5
Total (g)	100	100	100

CbR, rice enriched with transgenic Cblin pentapeptide; Non-Tg, non-transgenic rice.

Supplementary Table 3. Primer sequences.

Gene	Forward (5′→3′)	Reverse (5′→3′)
Mafbx	GGAAGCTTTCAACAGACTGGA	CTCAGGGATGTGAGCTGTGA
Murf1	ACGAGAAGAAGAGCGAGCTG	CTTGGCACTTGAGAGGAAGG
Actb	CACACCTTCTACAATGAGCTGC	CATGATCTGGGTCACTTTTCA

Supplementary Table 4. Mass spectrometry of CbR-specific signals in mouse serum.

m/z $[M + H]^+$	Predicted peptide sequence	RT (min)
710.2814	Gln-Asp-Gly-Tyr-Met-Pro	17.5
613.2286	Gln-Asp-Gly-Tyr-Met	14.6
482.1882	Gln-Asp-Gly-Tyr	15.5
582.2228	Asp-Gly-Tyr-Met-Pro	18.0
485.1701	Asp-Gly-Tyr-Met	14.2, 15.4

Values (*m/z*) correspond to predicted metabolites with ≥ 4 amino acid residues. CbR, rice enriched with transgenic Cblin pentapeptide; RT, retention time under LC-MS conditions described in legend to Supplementary Figure 2.
