# Supplementary material for: Oral intake of rice overexpressing ubiquitin ligase inhibitory pentapeptide prevents atrophy in denervated skeletal muscle
Source: NPJ Sci Food. 2021 Sep 9;5:25. doi: 10.1038/s41538-021-00108-0 (PMC8429733; doi:10.1038/s41538-021-00108-0)
Supplement: Supplementary file 2 — Reporting Summary [file 41538_2021_108_MOESM2_ESM.pdf]

nature research

Corresponding author(s): Takeshi Nikawa
Last updated by author(s): 2021/05/07

Reporting Summary

Nature Research wishes to improve the reproducibility of the work that we publish. This form provides structure for consistency and transparency in reporting. For further information on Nature Research policies, see our Editorial Policies and the Editorial Policy Checklist.

Statistics

For all statistical analyses, confirm that the following items are present in the figure legend, table legend, main text, or Methods section.

n/a	Confirmed	
☐	☒	The exact sample size (n) for each experimental group/condition, given as a discrete number and unit of measurement
☐	☒	A statement on whether measurements were taken from distinct samples or whether the same sample was measured repeatedly
☐	☒	The statistical test(s) used AND whether they are one- or two-sided<br>*Only common tests should be described solely by name; describe more complex techniques in the Methods section.*
☒	☐	A description of all covariates tested
☐	☒	A description of any assumptions or corrections, such as tests of normality and adjustment for multiple comparisons
☐	☒	A full description of the statistical parameters including central tendency (e.g. means) or other basic estimates (e.g. regression coefficient) AND variation (e.g. standard deviation) or associated estimates of uncertainty (e.g. confidence intervals)
☒	☐	For null hypothesis testing, the test statistic (e.g. F, t, r) with confidence intervals, effect sizes, degrees of freedom and P value noted<br>*Give P values as exact values whenever suitable.*
☒	☐	For Bayesian analysis, information on the choice of priors and Markov chain Monte Carlo settings
☒	☐	For hierarchical and complex designs, identification of the appropriate level for tests and full reporting of outcomes
☒	☐	Estimates of effect sizes (e.g. Cohen's d, Pearson's r), indicating how they were calculated

Our web collection on statistics for biologists contains articles on many of the points above.

Software and code

Policy information about availability of computer code

Data collection	Bruker Data Analysis software (version 3.2, Bruker Billerica); Chromaster System Manager (version 1.2, Hitachi Ltd.); StepOne software (version 2.3, Thermo Fisher Scientific Inc.)
Data analysis	Excel-Toukei 2015 software (version 3.21, Social Survey Research Information Co. Ltd.)

For manuscripts utilizing custom algorithms or software that are central to the research but not yet described in published literature, software must be made available to editors and reviewers. We strongly encourage code deposition in a community repository (e.g. GitHub). See the Nature Research guidelines for submitting code & software for further information.

Data

Policy information about availability of data

All manuscripts must include a data availability statement. This statement should provide the following information, where applicable:

- Accession codes, unique identifiers, or web links for publicly available datasets
- A list of figures that have associated raw data
- A description of any restrictions on data availability

The authors declare that the all data supporting the findings of this study are available within the paper and its supplementary information file.

Field-specific reporting

Life sciences study design

All studies must disclose on these points even when the disclosure is negative.

Sample size	No statistical method was used to determine the sample size for the experiments. The number of samples recorded in one group was chosen based on previous experience and standards in this field. Sample sizes were described in the manuscript.
Data exclusions	No data was excluded.
Replication	All experiments were reproduced multiple times in multiple cells or animal groups. All attempts of replication was successful.
Randomization	No randomization was performed as this is not strictly relevant to this study.
Blinding	Rice feeding experiments were performed blindly together with non-transgenic control rice.

Reporting for specific materials, systems and methods

We require information from authors about some types of materials, experimental systems and methods used in many studies. Here, indicate whether each material, system or method listed is relevant to your study. If you are not sure if a list item applies to your research, read the appropriate section before selecting a response.

Materials & experimental systems

n/a	Involved in the study	
☐	☒	Antibodies
☐	☒	Eukaryotic cell lines
☒	☐	Palaeontology and archaeology
☐	☒	Animals and other organisms
☒	☐	Human research participants
☒	☐	Clinical data
☒	☐	Dual use research of concern

Methods

n/a	Involved in the study	
☒	☐	ChIP-seq
☒	☐	Flow cytometry
☒	☐	MRI-based neuroimaging

Antibodies

Antibodies used	Commercially available antibodies were used. The primary antibodies were anti-IRS1 (06-248 Merck Millipore) and anti-ACTB (AC004 ABclonal Technology). Secondary antibodies were anti-rabbit IgG (GENA934 Cytiva) and anti-mouse IgG (GENA9310 Cytiva).
Validation	All antibodies are commercial in origin. Validation statements can be found on the manufacturer's website for the following:<br>anti-IRS1 (06-248 Merck Millipore): https://www.merckmillipore.com/JP/ja/product/Anti-IRS1-Antibody,MM_NF-06-248<br>anti-ACTB (AC004 ABclonal Technology): https://abclonal.co.jp/catalog-antibodies/ACTBMousemAb/AC004<br>anti-rabbit IgG (GENA934 Cytiva): https://www.sigmaaldrich.com/catalog/product/sigma/gena934100ul?lang=en&region=CA&gclid=CjwKCAjw19z6BRAYEiwAmo64Lb88-AEgaEzt70dpNziavWxnhZsYMhqAn6wOUdXuRhaXGQ4_u9hNcBoC4v8QAvD_BwE<br>anti-mouse IgG (GENA9310 Cytiva): https://www.sigmaaldrich.com/catalog/product/sigma/gena93101ml?lang=en&region=CA

Eukaryotic cell lines

Policy information about cell lines

Cell line source(s)	C2BBe1 clone of Caco-2 cells were obtained from ATCC (No. CRL-2102).
Authentication	No detection of HIV, HepB, HPV, EBV, and CMV by human pathogenic virus test
Mycoplasma contamination	All cell lines in our laboratory are routinely tested for mycoplasma contamination and cells used in this study are negative for mycoplasma.
Commonly misidentified lines (See ICLAC register)	No cell line used in the paper is listed in ICLAC database.

Animals and other organisms

Policy information about studies involving animals; ARRIVE guidelines recommended for reporting animal research

Laboratory animals	We used Sprague Dawley rats (6 weeks old) and ICR mice (6 weeks old). Animals were housed with access to diet and water ad libitum at 21 ± 1°C at Kyushu University and at 23 ± 2°C at Tokushima University under lights from 08:00 – 20:00.

Wild animals	The study did not involve wild animals.
Field-collected samples	The study did not involve field-collected samples.
Ethics oversight	All animal experiments proceeded according to the guidelines for animal experiments at Tokushima University and Kyushu University. The Ethics Review Committee for Animal Experimentation at these institutions approved all the experimental protocols, and thus were in accordance with NIH guidelines.

Note that full information on the approval of the study protocol must also be provided in the manuscript.
